# Endocrine Disruptors and Gynecological Malignancies

**DOI:** 10.3390/diagnostics16132116

**Published:** 2026-07-06

**Authors:** Dimitris Baroutis, Eleni Katsianou, Konstantinos Koukoumpanis, Ioannis Fragiskos, Nikolaos Sindos, Michael Sindos, George Daskalakis

**Affiliations:** 1 1st Department of Obstetrics and Gynecology, Alexandra Hospital, National and Kapodistrian University of Athens, 11528 Athens, Greecegdaskalakis@yahoo.com (G.D.); 2 2nd Department of Pediatrics, “P. & A. Kyriakou” Children’s Hospital, National and Kapodistrian University of Athens, 11527 Athens, Greece

**Keywords:** endocrine disruptors, gynecological malignancies, breast cancer, endometrial cancer, ovarian cancer, bisphenol A, phthalates, environmental carcinogens, biomarkers, risk stratification

## Abstract

**Background/Objectives:** Endocrine-disrupting chemicals (EDCs) interfere with hormonal homeostasis and have been implicated in gynecological malignancy pathogenesis. This narrative review synthesizes current evidence regarding EDC exposure and breast, endometrial, ovarian, and cervical cancers, examining molecular mechanisms, epidemiology, and diagnostic and clinical implications. **Methods:** We conducted a literature review using PubMed/MEDLINE, Embase, Scopus, and Cochrane databases through April 2026, including systematic reviews, meta-analyses, prospective cohorts, case-control studies, and mechanistic investigations examining EDC-cancer associations. Methodological quality was appraised using the Newcastle-Ottawa Scale and AMSTAR-2, with overall certainty of evidence rated using the GRADE framework. **Results:** Major EDC classes—bisphenol compounds, phthalates, polychlorinated biphenyls, organochlorine pesticides, and per- and polyfluoroalkyl substances—demonstrate carcinogenic potential through estrogen receptor modulation, epigenetic alterations, oxidative stress, and oncogenic signaling disruption. Breast cancer shows the strongest evidence, with prenatal and early-life DDT/DDE exposure associated with up to a 3.7-fold increased risk. Endometrial cancer demonstrates associations with xenoestrogen mixtures exhibiting non-monotonic dose-responses, whereas ovarian and cervical cancers show emerging but limited associations. Common mechanisms include receptor crosstalk, epigenetic dysregulation with transgenerational effects, oxidative genomic instability, metabolic reprogramming, and cancer stem cell enrichment. **Conclusions:** Evidence supports EDC contributions to gynecological malignancy through convergent pathways, though causal inference remains constrained by observational epidemiology, long latency periods, and challenges in characterizing real-world mixture exposures. Diagnostic and prevention strategies should integrate EDC exposure into risk-prediction models, leverage multi-omics biomarkers for early detection, and emphasize exposure reduction during critical developmental windows alongside regulatory reform.

## 1. Introduction

Gynecological malignancies represent a substantial component of the global cancer burden, with breast cancer alone accounting for 2.3 million new cases and 670,000 deaths in 2022, projected to double by 2040 [1]. Endometrial cancer incidence has increased by 132% in developed countries over the past three decades, particularly among younger women [2]. Ovarian cancer, while less common, carries the highest mortality among gynecological malignancies with five-year survival rates of only 45% [3]. While traditional risk factors including genetic predisposition, reproductive history, obesity, and hormonal exposures explain a substantial proportion of cases, they account for fewer than half of observed malignancies [4]. The rising incidence of early-onset estrogen receptor-positive breast cancer in women without identified genetic risk factors suggests environmental contributions, particularly from ubiquitous chemical exposures [5].

Endocrine-disrupting chemicals encompass a heterogeneous group of exogenous substances that interfere with hormone synthesis, secretion, transport, metabolism, binding, action, or elimination [6]. The Endocrine Society defines EDCs as exogenous chemicals or mixtures interfering with any aspect of hormone action [6]. These substances pervade modern environments through industrial processes, agricultural applications, consumer products, food packaging, personal care products, and building materials, with human exposure occurring via dietary ingestion, dermal absorption, inhalation, and in utero transfer [7]. The 2020 international consensus established 10 key characteristics of EDCs to enable systematic hazard identification, including hormone receptor interaction, alteration of receptor expression and signal transduction, induction of epigenetic modifications, and disruption of hormone synthesis, transport, distribution, metabolism, and target-cell fate [8].

The biological plausibility of EDC contributions to gynecological malignancy rests on several principles, though causal evidence varies across chemical classes and cancer types. First, breast, endometrial, and ovarian tissues demonstrate exquisite sensitivity to hormonal influences throughout the lifespan, with estrogens and progesterone regulating proliferation, differentiation, and apoptosis [9]. Second, vulnerability to endocrine disruption varies across the lifespan, with heightened susceptibility during preconception, fetal development, infancy, puberty, pregnancy, and perimenopause [10]. Third, EDCs exert biological effects at concentrations below traditional toxicological thresholds, often in parts-per-billion to parts-per-trillion ranges reflecting endogenous hormone activity [11]. Fourth, many EDCs show non-monotonic dose–response relationships in which low doses produce effects distinct from or greater than high doses [12]. Finally, human exposures invariably involve complex mixtures of dozens to hundreds of EDCs simultaneously, with potentially additive, synergistic, or antagonistic effects [13].

Scientific understanding of endocrine disruption has evolved dramatically over the past seven decades. Early observations in the 1940s documented reproductive abnormalities in pesticide-exposed wildlife. The diethylstilbestrol tragedy of the 1960s–1970s provided the first clear human evidence of developmental endocrine disruption causing cancer, linking prenatal DES exposure to clear cell adenocarcinoma of the vagina decades later [14]. The seminal 1991 Wingspread Conference catalyzed the modern EDC research field, and subsequent decades witnessed exponential growth in mechanistic understanding, epidemiological investigation, and regulatory attention, culminating in comprehensive assessments by the WHO, Endocrine Society, and European Commission [6,8].

Mechanistically, the association between EDC exposure and gynecological carcinogenesis is biologically coherent. Many EDCs are lipophilic xenoestrogens that bind the nuclear estrogen receptors (ERα and ERβ) and the membrane G-protein-coupled estrogen receptor (GPER), or that interfere with androgen, progesterone, and thyroid-hormone signaling, thereby mimicking or antagonizing endogenous hormones and driving unopposed proliferative signaling in hormone-responsive tissues [15,16,17]. Beyond direct receptor engagement, EDCs induce heritable epigenetic changes (DNA methylation, histone modification, and microRNA dysregulation) [18,19,20], generate reactive oxygen species that cause oxidative DNA damage and genomic instability [21,22], activate oncogenic signaling cascades (PI3K/AKT, MAPK/ERK, and STAT3) [23,24,25], and reprogram cellular metabolism and the inflammatory microenvironment [21,22,26,27,28]. Because hormone-sensitive gynecological tissues respond to even low-dose hormonal perturbation—particularly during prenatal and pubertal windows [9,10,11]—these convergent mechanisms provide a plausible biological basis for the epidemiological associations examined in this review; they are developed in detail in Section 4.

This literature review synthesizes current evidence linking EDC exposures to gynecological malignancies, with specific objectives to: classify major EDC categories and their sources; elucidate molecular mechanisms through which EDCs contribute to carcinogenesis; evaluate epidemiological evidence for associations between specific EDCs and breast, endometrial, ovarian, and cervical cancers; analyze dose–response relationships and critical exposure windows; assess clinical and diagnostic implications including biomarker development, integration into existing screening workflows, and incorporation into multifactorial risk-prediction models; identify knowledge gaps and future research priorities; and propose public health strategies for exposure mitigation and regulatory reform.

## 2. Methods

**Figure 1:**
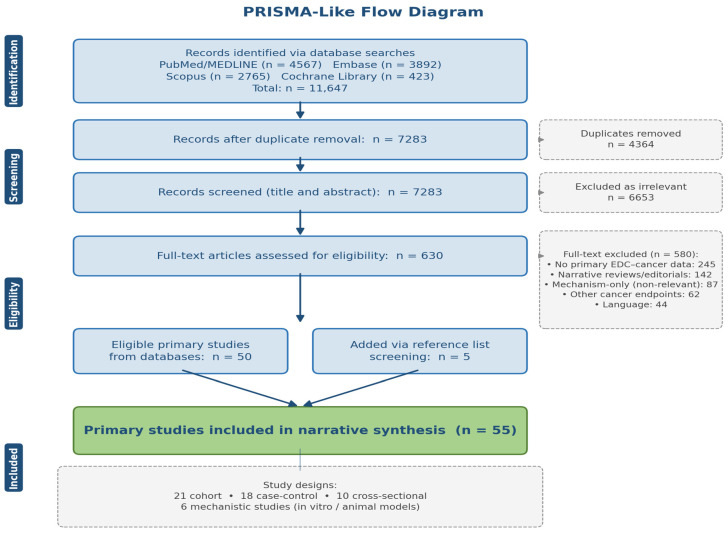
Flow diagram of the literature search and study selection process. Of 11,647 records identified across four databases, 55 primary EDC-cancer studies were included in the narrative synthesis (50 from database screening; 5 from reference list screening), comprising 21 cohort, 18 case-control, 10 cross-sectional, and 6 mechanistic studies. The manuscript additionally cites 55 contextual references for background pathophysiology, regulatory context, and methodological framework. Database-specific record counts reflect the results of the executed search strings in each database on the final search date (April 2026); the total reflects records before de-duplication.

This literature review was conducted in accordance with established methodological principles for evidence synthesis in environmental health and oncology. Although a disciplined search and selection framework was applied throughout, adherence to PRISMA reporting standards is not formally required for narrative reviews; however, in the interest of methodological transparency and reproducibility, a PRISMA-like flow diagram documenting the literature identification, screening, and inclusion process has been incorporated as Figure 1. Title and abstract screening, full-text eligibility assessment, and final inclusion decisions were performed independently by two reviewers (D.B. and E.K.), with disagreements resolved by consensus or, where necessary, by adjudication from a third reviewer (K.K.).

This article is a literature review and is not a systematic review nor a meta-analysis. A narrative format was deliberately chosen because the subject spans five major chemical classes, four distinct malignancies, and several levels of evidence—mechanistic, toxicological, epidemiological, and clinical—that are methodologically heterogeneous and therefore not amenable to quantitative pooling within a single meta-analytic framework. The structured search, explicit eligibility criteria, formal risk-of-bias and certainty appraisal (Newcastle-Ottawa Scale, AMSTAR-2, and GRADE), and PRISMA-style flow diagram described below were adopted to enhance the transparency, reproducibility, and rigor of the narrative synthesis, and not to support a statistical meta-analysis.

In the terminology of evidence synthesis, the present work is therefore best characterized as a structured literature (systematic-narrative hybrid) review: it combines the breadth and interpretive flexibility of a narrative review with a systematic search strategy and a structured appraisal step. The Newcastle-Ottawa Scale, AMSTAR-2, and GRADE were applied as transparent appraisal heuristics to communicate the relative certainty of the evidence and to avoid overstating weak associations; they are not presented as a formal systematic-review grading exercise, and no quantitative meta-analytic pooling was performed.

### 2.1. Search Strategy

Electronic searches covered four databases: PubMed/MEDLINE, Embase, Scopus, and the Cochrane Library. Search strings combined controlled vocabulary (MeSH/Emtree) with free-text terms across three concept blocks: EDC chemical classes (BPA, phthalates, PCBs, organochlorine pesticides, PFAS, parabens, heavy metals, dioxins) joined to mechanistic terms (endocrine disruption, hormonal carcinogenesis), gynecological cancer outcomes (breast, endometrial, ovarian, cervical neoplasms), and methodological filters (cohort, case-control, cross-sectional, mechanistic, and review designs). Searches covered January 2000 through April 2026, with reference list screening and citation tracking of pivotal articles to identify additional eligible studies.

### 2.2. Inclusion and Exclusion Criteria

Studies were included if they examined associations between defined EDC classes and breast, endometrial, ovarian, or cervical cancer in humans, or reported molecular mechanisms in biologically relevant experimental models. Eligible designs included systematic reviews and meta-analyses, prospective and retrospective cohort studies, case-control and cross-sectional studies with biomarker data, mechanistic studies in human cell lines or primary tissue using environmentally relevant concentrations, and authoritative consensus documents (WHO, Endocrine Society, European Commission, IARC). Studies were excluded if they examined non-gynecological endpoints exclusively, used exposure concentrations exceeding plausible human levels by more than three orders of magnitude without justification, were unavailable in English or Greek translation, or were retracted. The overall search and selection process is summarized in Figure 1.

No restriction was placed on participant age. Because EDC-related carcinogenesis reflects exposures accrued across the life course, eligible studies spanned the full age range represented in the source literature—from prenatal and early-life exposure cohorts, in which in-utero and childhood exposures were linked to malignancies arising decades later, through reproductive-age and peri- and post-menopausal women in whom the cancers were diagnosed. Where individual studies defined specific exposure windows or diagnostic age ranges, these are reported alongside the corresponding findings.

### 2.3. Evidence Hierarchy and Synthesis

A narrative synthesis approach was selected as methodologically appropriate for this breadth of evidence spanning chemistry, toxicology, molecular biology, epidemiology, and clinical medicine. This approach enables integration of heterogeneous evidence types that cannot be pooled through meta-analysis—including mechanistic, toxicological, and epidemiological data—while allowing explicit critical appraisal of individual study quality. Evidence sources were prioritized in descending order: (1) systematic reviews and meta-analyses; (2) prospective cohort studies with biomarker-confirmed exposure; (3) large case-control studies with biomarker data; (4) cross-sectional and smaller case-control studies; (5) mechanistic studies at environmentally relevant concentrations; and (6) expert consensus statements. The structured search strategy, explicit inclusion criteria, formal quality appraisal, and PRISMA-like flow diagram (Figure 1) provide methodological transparency.

By primary cancer focus, the 55 included primary studies comprised 23 addressing breast cancer—reflecting the largest and most mature evidence base—14 addressing endometrial cancer, 10 addressing ovarian cancer, and 8 addressing cervical cancer; studies that addressed more than one malignancy were assigned to their principal outcome, and the six mechanistic studies were allocated to the cancer type they primarily modelled. This distribution mirrors the depth of evidence presented in Section 5, Section 6, Section 7 and Section 8, in which breast cancer carries the strongest epidemiological support and cervical cancer the most limited.

In synthesizing this evidence, we explicitly distinguish three tiers of inference and avoid conflating them. Mechanistic evidence—derived predominantly from in vitro systems and animal models—establishes biological plausibility and candidate pathways but cannot, on its own, demonstrate human carcinogenicity. Associative evidence from observational human studies (cohort, case-control, and cross-sectional designs) can establish exposure–outcome correlations but remains vulnerable to confounding, reverse causation, and exposure misclassification. Causal evidence, which would require triangulation across consistent, temporally appropriate, and dose-dependent human data supported by coherent mechanisms, is at present limited for most EDC-gynecological-cancer relationships. Accordingly, the associations reported throughout this review should be read as associative and hypothesis-consistent rather than as established causal relationships, except where explicitly qualified, and the directional schema “exposure → pathway activation → malignancy” should be understood as a mechanistic hypothesis rather than a demonstrated causal chain.

### 2.4. Quality Assessment

A structured methodological quality appraisal was applied to all primary studies informing key conclusions. Observational studies (cohort and case-control designs) were appraised using the Newcastle-Ottawa Scale (NOS), evaluating selection (≤4 stars), comparability (≤2 stars), and outcome/exposure ascertainment (≤3 stars); studies scoring ≥ 7 stars were classified as low risk of bias, 5–6 as moderate, and <5 as high. Systematic reviews and meta-analyses were appraised using AMSTAR-2. Overall certainty of evidence for key epidemiological conclusions was assessed using GRADE, classifying evidence as High, Moderate, Low, or Very Low based on study design, risk of bias, inconsistency, indirectness, imprecision, and publication bias. Given that the evidence base is predominantly retrospective, subject to exposure assessment limitations from single-time-point biomarkers for long-latency diseases, and affected by residual confounding from mixture co-exposures, overall GRADE certainty was rated Low to Moderate for breast cancer (strongest evidence) and Very Low to Low for endometrial, ovarian, and cervical cancer. Mechanistic evidence from in vitro and animal studies was appraised for biological plausibility, dose relevance, and reproducibility but not formally GRADE-rated, consistent with standard practice.

## 3. Classification and Sources of Endocrine-Disrupting Chemicals

Table 1 provides a comprehensive classification of major EDC categories relevant to gynecological malignancy risk.

Bisphenol A is the most extensively studied EDC, produced at over 8 million metric tons annually for polycarbonate plastic and epoxy resin manufacture. Human exposure occurs primarily through dietary ingestion (~93% of total) from BPA leaching out of food containers, beverage bottles, and can linings, with leaching increased by heating, acidic content, and container age [29]. The 2003–2004 NHANES survey detected BPA in 93% of urine samples from a representative US population (geometric mean 2.6 ng/mL), indicating widespread continuous exposure [29]. Regulatory concerns prompted industrial substitution with structural analogues including bisphenol S, F, and AF, marketed as “BPA-free” alternatives, though accumulating evidence demonstrates comparable or greater endocrine-disrupting potency for several substitutes [30,31].

Phthalates function as plasticizers added to polyvinyl chloride products and as solvents in personal care products, cosmetics, and pharmaceuticals, with human exposure ubiquitous and multi-route, including dietary ingestion from contaminated foods, dermal absorption from personal care product application, and inhalation of contaminated indoor air and dust [32]. The 2007–2008 NHANES detected multiple phthalate metabolites in over 95% of participants, with particularly high concentrations among women of reproductive age using fragranced products, cosmetics, and hair care products [33]. Di(2-ethylhexyl) phthalate represents the most produced phthalate at approximately 2 million metric tons annually, with primary uses in vinyl flooring, medical devices, and food packaging [34].

Polychlorinated biphenyls, despite manufacturing prohibition in 1979, persist in the environment due to extreme chemical stability and bioaccumulation in food chains. Human exposure occurs primarily through dietary consumption of contaminated fish, meat, and dairy products, accounting for over 90% of intake, with higher-chlorinated congeners accumulating in adipose tissue with half-lives measured in years to decades [35]. A 2016 meta-analysis including 23 studies and over 12,000 breast cancer cases demonstrated positive associations between PCB exposures and breast cancer risk with a pooled relative risk of 1.37 (95% CI 1.12–1.68) for highest versus lowest exposure categories [36].

Organochlorine pesticides including DDT, DDE, chlordane, dieldrin, and lindane were widely used from the 1940s through the 1970s for agricultural pest control and malaria vector management before being banned in most developed countries due to environmental persistence and bioaccumulation [37]. However, DDT continues in use for malaria control in endemic regions under World Health Organization exemptions, and historical contamination ensures ongoing human exposure through consumption of contaminated foods, particularly animal products with adipose tissue accumulation [38]. DDE, representing DDT’s major metabolite, demonstrates greater persistence and estrogenicity than the parent DDT compound.

Per- and polyfluoroalkyl substances encompass over 4700 synthetic chemicals characterized by carbon-fluorine bonds conferring exceptional stability and resistance to degradation. Major PFAS, including PFOA and PFOS, demonstrate environmental persistence measured in decades, bioaccumulation in humans with serum half-lives of 2–9 years, and contamination of drinking water supplies affecting millions globally [39,40]. PFAS applications span food packaging, non-stick cookware, stain-resistant textiles, firefighting foams, and industrial processes, with human exposure occurring through contaminated drinking water, dietary intake, occupational exposures, and consumer product use [39,40,41].

Heavy metals (cadmium, arsenic, lead) act as metalloestrogens through estrogen receptor activation at nanomolar concentrations. Cadmium exposure occurs primarily through cigarette smoke, contaminated foods (rice, polluted-soil vegetables), and occupational settings, with a 10–30-year biological half-life ensuring chronic accumulation [42]. Inorganic arsenic exposure occurs mainly via contaminated drinking water in endemic regions (Bangladesh, India, Taiwan, Chile) and rice consumption, with mechanisms including oxidative stress, epigenetic modifications, and hormonal disruption [43,44]. Parabens serve as preservatives in cosmetics, pharmaceuticals, and food, with urinary concentrations of 1–1000 ng/mL despite relatively weak estrogenic activity [45,46].

## 4. Molecular Mechanisms of EDC-Mediated Carcinogenesis

**Figure 2:**
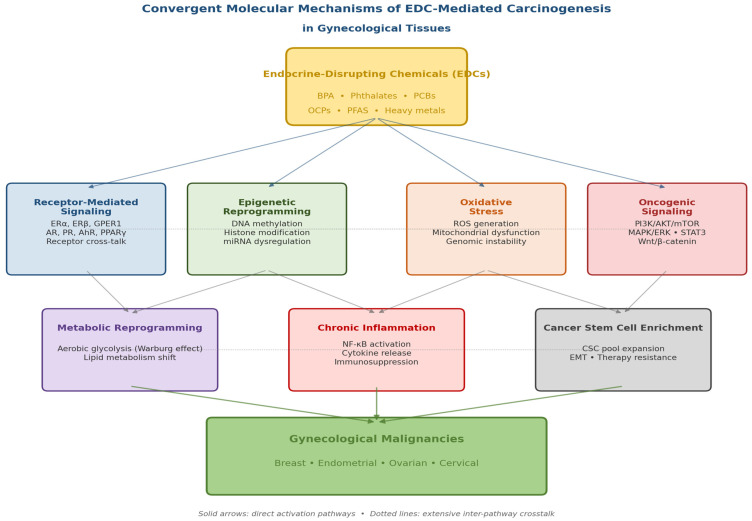
Convergent molecular mechanisms of EDC-mediated carcinogenesis in gynecological tissues. EDCs activate multiple interconnected pathways including estrogen receptor signaling (ERα, ERβ, GPER1), epigenetic reprogramming (DNA methylation, histone modifications, miRNA dysregulation), oxidative stress-mediated genomic instability, oncogenic signaling cascades (PI3K/AKT, MAPK/ERK, STAT3, Wnt/β-catenin), metabolic reprogramming toward aerobic glycolysis, chronic inflammatory microenvironment, and cancer stem cell enrichment. These pathways exhibit extensive crosstalk and tissue-specific activation patterns across breast, endometrial, ovarian, and cervical tissues.

For clarity and readability, the mechanisms discussed below are organized into four interrelated categories: (i) receptor-mediated effects (Section 4.1); (ii) epigenetic and transgenerational effects (Section 4.2); (iii) oxidative and metabolic dysregulation (Section 4.3 and Section 4.4); and (iv) oncogenic signaling and cancer stem cell alterations (Section 4.4). These categories are not mutually exclusive but interact extensively, as summarized in Table 2 and illustrated in Figure 2.

Table 2 summarizes the major molecular mechanisms through which EDCs contribute to gynecological cancer pathogenesis.

### 4.1. Receptor-Mediated Mechanisms

Estrogen receptors function as ligand-activated transcription factors regulating cell proliferation, differentiation, and survival. EDCs interact with these receptors through direct binding, non-genomic signaling, and selective receptor modulation [15]. BPA binds ERα and ERβ with 1000–10,000-fold lower affinity than 17β-estradiol but achieves biological activity at environmental concentrations through receptor conformational changes favoring coactivator recruitment, preferential ERβ activation, membrane-initiated signaling via GPER1, and extended receptor occupancy due to slow dissociation [16]. Many EDCs activate rapid, non-genomic ER signaling pathways including MAPK/ERK (proliferation), PI3K/AKT (survival/metabolism), and calcium mobilization [17].

Beyond estrogen receptors, EDCs interact with multiple hormone receptor systems. Phthalates demonstrate anti-androgenic properties through competitive receptor antagonism and reduced testosterone synthesis, disrupting estrogen/androgen balance critical for breast tissue homeostasis [18]. Parabens bind progesterone receptors with moderate affinity, potentially disrupting protective progesterone effects in breast and endometrial tissues. PCBs, PFAS, and flame retardants interfere with thyroid hormone synthesis, transport, and receptor activation, indirectly affecting cancer risk through altered estrogen metabolism [19]. Dioxins and dioxin-like PCBs activate the aryl hydrocarbon receptor, inducing CYP1A1 and altering estrogen metabolism while promoting inflammatory responses [20]. Phthalates activate PPARγ, influencing adipogenesis and contributing to obesity-associated cancer risk [50].

### 4.2. Epigenetic Mechanisms

Epigenetic modifications represent heritable changes in gene expression without alterations in DNA sequence, providing mechanistic links between developmental EDC exposure and latent cancer manifestation decades later. EDCs induce aberrant DNA methylation patterns in promoter regions, CpG islands, and intergenic regions through global hypomethylation that potentially activates proto-oncogenes and promotes genomic instability, as well as gene-specific hypermethylation wherein tumor suppressor genes including BRCA1, p16INK4a, and RASSF1A undergo hypermethylation following developmental EDC exposure, silencing protective pathways [51]. Imprinted genes such as H19, IGF2, and MEG3 that regulate growth and differentiation become disrupted by EDC exposure [52].

Post-translational histone modifications including acetylation, methylation, phosphorylation, and ubiquitination regulate chromatin structure and gene transcription. BPA increases histone acetyltransferase activity while decreasing histone deacetylase activity, resulting in hyperacetylated chromatin states favoring gene transcription [53]. Cadmium disrupts histone methyltransferase and demethylase balance, altering H3K9me3 and H3K27me3 marks associated with gene silencing [54]. MicroRNAs represent small non-coding RNAs regulating post-transcriptional gene expression, with EDCs inducing widespread miRNA dysregulation, including downregulation of tumor suppressor miRNAs such as miR-21 [54,55], miR-200 family, and let-7 following BPA exposure, as well as upregulation of oncogenic miRNAs including miR-155 and miR-221 promoting proliferation and anti-apoptotic signaling [55,56].

### 4.3. Oxidative Stress and Genomic Instability

Reactive oxygen species generation is a common consequence of EDC exposure across chemical classes, arising from heavy-metal-catalyzed Fenton reactions, mitochondrial dysfunction, NADPH oxidase activation, and depletion of glutathione, superoxide dismutase, and catalase [21]. Oxidative DNA damage manifests as 8-oxo-7,8-dihydroguanine (the most abundant and highly mutagenic oxidative lesion), single- and double-strand breaks promoting chromosomal rearrangements, and base modifications including thymine glycol and 8-oxoadenine [22]. Lipid peroxidation generates reactive aldehydes (malondialdehyde, 4-hydroxynonenal) forming DNA adducts and protein modifications, while protein oxidation impairs DNA repair enzymes, cell cycle checkpoints, and antioxidant defenses, creating vicious cycles of cumulative damage [21].

### 4.4. Oncogenic Signaling Pathways

EDCs activate multiple interconnected oncogenic signaling cascades promoting proliferation, survival, and metastasis. The PI3K/AKT pathway, regulating cell survival, metabolism, and protein synthesis, is activated by BPA, phthalates, and PFAS through receptor tyrosine kinase engagement, promoting glycolytic enzyme expression and inhibiting pro-apoptotic proteins (BAD, caspase-9) [24,25]. MAPK/ERK signaling promotes proliferation through c-Fos, c-Jun, and c-Myc activation [23]. STAT3 activation drives proliferation, angiogenesis, and immune evasion via cyclin D1, Bcl-xL, and VEGF induction, creating immunosuppressive microenvironments and promoting epithelial-mesenchymal transition [25].

Although aerobic glycolysis (the Warburg effect) is the most frequently cited metabolic alteration [57], EDC-driven metabolic reprogramming is not confined to glycolysis. Environmentally relevant exposures to bisphenols, phthalates, and cadmium have also been reported to enhance glutaminolysis that replenishes tricarboxylic-acid-cycle intermediates, to promote de novo lipogenesis and fatty-acid oxidation that supply membrane lipids and redox cofactors, to remodel mitochondrial oxidative phosphorylation and dynamics, to divert glucose into the pentose phosphate pathway for nucleotide and NADPH production, and to engage one-carbon (folate) metabolism that supports the aberrant DNA-methylation patterns described in Section 4.2 [21,22,26,27,28]. Glycolysis is therefore best regarded as the most extensively documented node within a broader, interconnected metabolic rewiring that collectively sustains the biosynthetic and redox demands of EDC-exposed proliferating cells.

As illustrated in Figure 2, these molecular mechanisms converge and exhibit extensive crosstalk across gynecological tissues, creating multiple therapeutic opportunities for prevention and intervention through targeting shared pathways that drive EDC-mediated carcinogenesis.

Across the four malignancies examined below, the strength of human evidence differs markedly, and we grade it into four explicit tiers that correspond to the GRADE certainty ratings applied in this review. The evidence is strongest for breast cancer, where prospective cohort data link early-life DDT/DDE exposure and several polychlorinated biphenyl congeners to increased risk (strong evidence; Moderate GRADE certainty) [26,36,58,59]. It is moderate for endometrial cancer, resting largely on xenoestrogen-mixture and occupational-exposure studies (moderate evidence; Low-Moderate certainty) [60,61]. It is emerging for ovarian cancer, where associations are biologically plausible but sparse and inconsistent (emerging evidence; Low certainty) [62,63]. It is hypothesis-level for cervical cancer, where any EDC contribution is at most a cofactor to human papillomavirus infection and is supported chiefly by mechanistic reasoning (hypothesis-level evidence; Very Low certainty) [64,65]. The cancer-specific sections that follow should be weighed against these tiers.

## 5. Breast Cancer: Strongest Epidemiological Evidence

Table 3 summarizes major epidemiological studies examining EDC-breast cancer associations.

Extensive epidemiological investigation has examined DDT and DDE associations with breast cancer. Studies emphasizing age at exposure have demonstrated that early-life DDT exposure carries substantially greater breast cancer risk than adult exposure, with elevated relative risks observed across multiple cohorts [58]. The Child Health and Development Studies prospective cohort measured maternal serum DDT during 1959–1967 pregnancies and followed daughters: women exposed to p,p′-DDT before age 14 exhibited 3.7-fold increased breast cancer risk (95% CI 1.5–9.2), with strongest associations for ER-positive tumors [26] (GRADE: Moderate). The Long Island Breast Cancer Study Project (1508 cases, 1556 controls) found elevated DDE associations among women born after 1931, with OR 1.44 (95% CI 1.02–2.05) for highest versus lowest quintile [59].

PCB-breast cancer relationships demonstrate complex congener-specific patterns (GRADE certainty: Low for individual congener associations). Congeners with estrogenic properties, including PCB-52, PCB-187, and PCB-203, show positive associations with pooled odds ratios ranging from 1.3 to 1.6 for highest versus lowest exposure, while dioxin-like PCBs including PCB-126 and PCB-169 demonstrate weaker or null associations potentially reflecting competing AhR-mediated anti-estrogenic effects [36]. The CHDS cohort demonstrated that PCB exposure measured shortly after giving birth was associated with a 1.3-fold increased risk of breast cancer before age 50 years (HR 1.27; 95% CI 1.00–1.61) [36,71]. These associations should be interpreted in light of substantial between-study heterogeneity and the potential for publication bias. The pooled meta-analytic estimate [36] was accompanied by moderate-to-high statistical heterogeneity (reported I^2^ values exceeding 50% for several congeners), attributable to differences in exposure assessment (serum versus adipose measurement, lipid adjustment), timing of biospecimen collection relative to the latency window, congener-specific quantification, and variation in confounder control across cohorts such as the CHDS and Long Island studies. Smaller positive studies may be over-represented in the literature, and funnel-plot asymmetry consistent with publication bias has been noted in pooled analyses of organochlorine-breast cancer associations; the individual cohort findings presented above are therefore best regarded as hypothesis-consistent rather than confirmatory and are reflected in the Low-GRADE certainty assigned to congener-specific estimates.

BPA epidemiological evidence remains mixed despite extensive in vitro and animal data, with overall GRADE certainty rated Very Low to Low, with challenges including BPA’s short 4–6 h half-life necessitating repeated measurements to characterize chronic exposure, near-universal BPA detection limiting exposure contrast and reducing statistical power, and study heterogeneity [6]. Recent advances using novel approaches show emerging positive findings, with breast adipose tissue BPA concentrations compared to urinary biomarkers correlating with breast cancer risk (OR 1.8; 95% CI 1.1–3.0), and genetic polymorphisms in BPA-metabolizing enzymes modifying associations with slow metabolizers showing elevated risk [66,67,72].

The Multiethnic Cohort case-control study including 802 cases and 793 controls demonstrated positive associations between DEHP metabolite concentrations and breast cancer risk, particularly for hormone receptor-positive disease, with mono(2-ethylhexyl) phthalate (OR 1.45; 95% CI 1.12–1.89) for the highest versus lowest quartile [68]. Associations proved strongest among premenopausal women. A Mexican case-control study identified diethyl phthalate associations with breast cancer (OR 2.2; 95% CI 1.3–3.6) for the highest versus lowest tertile, particularly among premenopausal women with high body mass index [69].

BPA activates ER-dependent transcription at concentrations as low as 0.01–1 nM despite a 1000-fold lower binding affinity than 17β-estradiol. Mechanisms underlying this potency include preferential ERβ activation (~10-fold over ERα), high-affinity GPER1 activation triggering rapid non-genomic signaling, and coactivator recruitment via receptor conformational changes [73]. Developmental BPA exposure permanently alters mammary architecture: increased ductal density, branching, and terminal end bud numbers following prenatal/perinatal exposure; expanded mammary stem and progenitor cell populations with heightened proliferative capacity; and disrupted stroma-epithelium signaling, including altered collagen deposition and growth factor expression [74]. These perturbations establish susceptibility windows in which subsequent hormonal exposures or spontaneous mutations more readily promote tumorigenesis.

Developmental BPA exposure induces hypermethylation of tumor suppressor genes (BRCA1, RASSF1A, p16) and hypomethylation of oncogenes and repetitive elements, with marks persisting into adulthood and predisposing to malignant transformation [75]. BPA downregulates tumor suppressor microRNAs (let-7, miR-200 family) while upregulating oncogenic microRNAs (miR-21, miR-155); miR-200 suppression promotes epithelial-mesenchymal transition and invasive phenotypes [76]. A landmark 2025 study demonstrated that BPA and DDT disrupt mammary adipocyte function, creating pro-tumorigenic microenvironments through adipocyte dedifferentiation (reduced adiponectin, increased leptin and inflammatory cytokines), aromatase induction elevating local estrogen biosynthesis, and metabolic inflammation with macrophage recruitment and IL-6, TNF-α, and PGE2 secretion establishing chronic inflammatory microenvironments [67].

The estrogen window hypothesis posits that breast cancer risk reflects cumulative estrogen exposure duration. EDC exposures during specific life stages exert disproportionate effects: in utero animal exposure increases mammary cancer susceptibility 100-fold compared with adult exposure, while DES daughters demonstrate 40-fold elevated clear cell adenocarcinoma risk [77]. Pre-menarchal exposure affects breast cancer risk more strongly than post-pubertal exposures in DDT-exposed cohorts. Pubertal exposure during rapid ductal elongation creates vulnerability, with phthalate exposure correlating with increased mammographic density. Pregnancy is both protective (terminal differentiation) and vulnerable (extensive proliferation), with EDC timing determining net effects [78].

## 6. Endometrial Cancer: Mechanisms and Evidence

Endometrial cancer exhibits strong hormone dependence, rendering it susceptible to endocrine disruption, with Type I cancers (80% of cases) arising from prolonged unopposed estrogen stimulation. A landmark 2024 Environmental Health Perspectives study investigated EDC mixtures and endometrial cancer risk in over 300 Spanish and Catalan women, finding that the highest xenoestrogen exposure quartile conferred 2.1-fold increased risk (95% CI 1.3–3.4) after adjustment for BMI, parity, and oral contraceptive use [61]. Notably, second- and third-quartile exposures showed stronger associations (OR 2.5–2.8) than the highest quartile (OR 2.1), supporting non-monotonic relationships in which intermediate concentrations exert maximal effects (GRADE: Low). This apparent non-monotonic pattern, however, must be interpreted cautiously, as alternative explanations cannot be excluded. The overlapping confidence intervals across the upper exposure quartiles indicate that the difference between the moderate-exposure and highest-exposure estimates may reflect chance (sampling variability) rather than a true biological inversion, particularly given the modest sample size (just over 300 participants), which yields wide interval estimates and limited statistical power for quartile-specific contrasts. Residual confounding provides a further plausible explanation: incomplete adjustment for adiposity (BMI is an imperfect surrogate for the adipose compartment in which lipophilic xenoestrogens accumulate and where aromatase-driven estrogen biosynthesis occurs), dietary and lifestyle correlates of exposure, and competing-risk and selection effects could each generate a spurious inverted-U shape. Exposure misclassification from single time-point biomarker measurement for a long-latency disease may additionally distort the dose–response shape. Accordingly, while these findings are consistent with the non-monotonic behavior reported in experimental models, they should be regarded as hypothesis-generating; confirmation requires larger cohorts with repeated biomarker measurement and formal modelling of the exposure–response curve.

Alkylphenols including nonylphenol and octylphenol function as xenoestrogens with approximately one-thousandth the potency of 17β-estradiol. A 2020 case-control study measuring urinary alkylphenol concentrations found that women with endometrial cancer exhibited 2.4-fold higher urinary nonylphenol levels compared to controls, with a median of 14.3 versus 6.1 ng/mL, and the highest nonylphenol tertile associated with a 3.1-fold increased risk (95% CI 1.7–5.6) after multivariable adjustment [60]. Associations proved strongest for Type I endometrioid adenocarcinomas (OR 3.8) compared with Type II non-endometrioid cancers (OR 1.4, not statistically significant).

Agricultural workers face elevated pesticide exposures, with multiple studies demonstrating endometrial cancer associations. The Spanish Screenwide case-control study (527 cases, 578 controls) identified occupational organophosphate exposure associated with a 1.6-fold increased risk (95% CI 1.1–2.3), legacy pesticide exposure (DDT, lindane) with a 1.8-fold elevated risk (95% CI 1.2–2.7), and current-use synthetic pyrethroids with weaker associations (OR 1.2; 95% CI 0.9–1.7) [61]. NHANES analysis of PFAS-cancer associations found women with prior uterine cancer exhibited significantly elevated PFNA concentrations versus women without cancer history (geometric means 1.8 vs. 1.2 ng/mL) [77].

A comprehensive 2022 systematic review synthesized mechanistic evidence from 25 studies examining EDC effects on endometrial cancer pathways. BPA, phthalates, and alkylphenols activate ERα in endometrial epithelial cells at environmentally relevant concentrations from 0.1 to 10 nM, inducing proliferative gene expression including cyclin D1, c-Myc, and IGF-1 [24]. Several EDCs demonstrate anti-progestogenic properties antagonizing progesterone’s protective effects, with parabens competing with progesterone for receptor binding and PCBs reducing progesterone receptor expression, disrupting the estrogen-progesterone balance critical for endometrial homeostasis [79]. BPA promotes endometrial epithelial cell proliferation while inhibiting apoptosis through Bcl-2 upregulation, representing anti-apoptotic signaling; Bax downregulation, representing pro-apoptotic signaling; and caspase-3 activity suppression.

Polycyclic aromatic hydrocarbons, representing products of incomplete combustion ubiquitous in grilled meats, tobacco smoke, and air pollution, exert endometrial cancer-promoting effects through AhR activation. AhR activation induces cytochrome P450 1A1, producing reactive PAH metabolites forming DNA adducts and altered estrogen metabolism favoring 4-hydroxyestrone, representing a genotoxic metabolite over 2-hydroxyestrone, representing a non-genotoxic metabolite [80]. AhR-mediated COX-2 induction increases PGE2 production promoting proliferation and angiogenesis, while AhR interacts with ERα modulating estrogen-responsive gene expression and potentially amplifying estrogenic EDC effects [81].

Cadmium exerts multiple pro-carcinogenic effects in endometrial tissue through estrogen receptor activation by binding ERα at distinct sites from endogenous estrogens inducing conformational changes that activate transcription, aromatase induction increasing CYP19 expression elevating local estrogen biosynthesis, oxidative stress generating ROS while depleting glutathione causing oxidative DNA damage and lipid peroxidation, epigenetic alterations disrupting DNA methyltransferase activity inducing global hypomethylation and gene-specific hypermethylation patterns, and exceptionally long half-life of 10-30 years ensuring chronic tissue exposure even after exposure cessation facilitating cumulative carcinogenic effects [82].

A landmark 2023 NIEHS study in mice demonstrated that neonatal diethylstilbestrol exposure establishes endometrial adenocarcinoma predisposition through developmental reprogramming. Developmental exposure (first hit) permanently altered uterine epithelial differentiation through Wnt/β-catenin pathway activation, PI3K/AKT hyperactivation, altered chromatin accessibility at proliferation-related gene promoters, and expansion of stem and progenitor cell populations with heightened transformation susceptibility [26]. Adult exposure (second hit) in developmentally primed uteri readily induced adenocarcinoma, whereas mice without developmental exposure resisted tumor formation despite identical adult exposures. DES daughters demonstrate 5-fold elevated endometrial cancer risk with 30–40-year latency, providing compelling human evidence for developmental origins of endometrial cancer [14].

## 7. Ovarian Cancer: Emerging Evidence and Mechanisms

Ovarian cancer epidemiology presents unique challenges for evaluating EDC associations given the ovary’s dual endocrine and reproductive roles combined with cancer heterogeneity. The USC/UCSF NHANES analysis from 2005–2018 examining EDC biomarkers in women with and without prior cancer diagnoses found significant associations for ovarian cancer, with perfluorononanoic acid showing 1.8-fold higher concentrations in ovarian cancer survivors with geometric mean 1.9 versus 1.1 ng/mL, perfluorodecanoic acid demonstrating 1.6-fold elevation, and PFOS showing borderline significant elevation [77]. Among phenols, 2,5-dichlorophenol showed 2.1-fold higher levels in the ovarian cancer group, bisphenol A demonstrated 1.4-fold elevation, and benzophenone-3, representing a sunscreen ingredient, showed 1.7-fold elevation.

Occupational and residential pesticide exposure studies demonstrate inconsistent ovarian cancer associations, with trans-nonachlor (an organochlorine insecticide) showing OR 1.7 (95% CI 1.1–2.6) for highest versus lowest quartile in agricultural workers, and dioxins showing elevated ovarian cancer incidence in the Seveso cohort following industrial accident [62]. DDT and DDE in most general population studies show null associations, as do current-use pesticides in agricultural health studies.

A 2025 computational network toxicology study identified 234 molecular targets linking phthalates to ovarian cancer through pathway analysis and molecular docking, with core target genes including GAPDH regulating glycolysis, CASP3 regulating apoptosis, PPARG regulating adipogenesis and metabolism, ESR1 representing estrogen receptor α, and CYCS regulating mitochondrial apoptosis [63]. Pathway enrichment showed HIF-1 signaling pathway, PI3K/AKT pathway, MAPK pathway, and estrogen signaling pathway with significant enrichment, suggesting multiple convergent mechanisms.

A 2025 landmark study demonstrated that BPA induces ovarian cancer stem cells in mice through oxidative stress-mediated mechanisms, with cancer stem cell expansion showing a 3-fold increase in CD44-positive CD133-negative populations, a 5-fold increase in CD44-positive CD133-positive double-positive populations, enhanced tumorsphere formation capacity, and increased expression of stemness markers including Oct4, Sox2, and Nanog [25]. Oxidative stress manifested as elevated ROS production showing a 2.5-fold increase, decreased antioxidant gene expression with SOD1 showing a 40% reduction, SOD2 showing a 55% reduction, CAT showing a 45% reduction, GPX1 showing a 50% reduction, and FOXO3 showing a 35% reduction, alongside oxidative DNA damage through 8-oxoGua adducts. BPA-induced ROS activates NF-κB and Wnt/β-catenin signaling, promoting stem cell self-renewal and survival, with antioxidant supplementation using *N*-acetylcysteine abrogating BPA effects, confirming oxidative stress mediation.

A 2025 systems biology investigation identified CXCL8 representing IL-8 and MMP9 as key genes upregulated in ovarian, endometrial, and cervical cancer cells following BPA exposure at environmentally relevant concentrations of 0.5 nM. CXCL8 showed 4-fold upregulation in ovarian cancer cells, promoting neutrophil and macrophage recruitment, creating inflammatory microenvironments, with autocrine signaling through CXCR1 and CXCR2 enhancing migration and invasion associated with advanced stage, metastasis, and poor prognosis [25]. MMP9 demonstrated 3-fold upregulation, degrading extracellular matrix components including collagen IV and gelatin, facilitating invasion, promoting angiogenesis through VEGF release from matrix stores, and correlating with peritoneal dissemination patterns. Invasion assays showed BPA-treated cells demonstrated 2.5-fold increased invasion through Matrigel-coated membranes blocked by CXCR1/2 or MMP9 inhibitors.

Low-dose BPA from 0.01 to 1 nM stimulates ovarian cancer cell proliferation by enhancing glycolytic metabolism through ER-dependent mechanisms, with 2-fold increased glucose consumption and lactate production, upregulation of glycolytic enzymes including hexokinase-2, phosphofructokinase, and lactate dehydrogenase A, and a paradoxical increase in ATP despite reduced oxidative phosphorylation, reflecting glycolytic compensation representing the Warburg effect [57]. Enhanced glycolysis correlates with proliferation rate, chemotherapy resistance, and tumor aggressiveness. Perfluorooctanoic acid disrupts ovarian Hippo signaling, impairing folliculogenesis and potentially promoting transformation, with PFOA reducing LATS1/2 phosphorylation activating YAP, nuclear YAP accumulation driving TEAD-mediated transcription of proliferative and anti-apoptotic genes, and loss of contact inhibition with enhanced anchorage-independent growth [49].

## 8. Cervical Cancer: Limited Evidence and HPV Cofactor Hypotheses

Cervical cancer differs fundamentally from other gynecological malignancies in having a well-established infectious etiology with high-risk human papillomavirus representing the necessary causal agent. HPV types 16 and 18 account for 70% of cervical cancers globally, though only a small fraction, from 0.3 to 1% of women with persistent high-risk HPV infection, progress to invasive cancer, suggesting cofactor involvement [64]. The two-hit model for cervical carcinogenesis requires the first hit representing persistent high-risk HPV infection, followed by the second hit representing additional insults promoting viral oncogene E6 and E7 expression, immune evasion, or genomic instability, with EDCs potentially functioning as second-hit cofactors, though evidence remains limited [83].

The strongest epidemiological evidence links cadmium to cervical neoplasia through mechanisms including cervical tissue accumulation at levels 3-fold higher than serum, immune suppression impairing cervical immune surveillance potentially facilitating HPV persistence through reduced Langerhans cell density in cervical epithelium, decreased natural killer cell cytotoxicity, and impaired T cell proliferation and cytokine production, as well as oxidative DNA damage generating ROS causing DNA strand breaks and oxidative base modifications potentially synergizing with HPV E6/E7-induced genomic instability [84]. Cigarette smoking represents the major cadmium exposure source and an established independent cervical cancer risk factor, with disentangling cadmium’s direct effects from smoking-related confounding proving challenging as most studies inadequately control for smoking intensity, duration, and pack-years [85]. Endemic arsenic exposure regions including Bangladesh and Taiwan demonstrate elevated cervical cancer incidence with proposed mechanisms including HPV immune evasion promotion and enhanced E6/E7 expression, though data remain limited [86].

EDCs may promote persistent HPV infection through epithelial differentiation effects wherein normal cervical epithelial differentiation limits HPV replication to basal layers while BPA disrupts epithelial differentiation programs, potentially expanding HPV-permissive cell populations, as well as local immune suppression wherein cadmium and other heavy metals impair cervical tissue immune responses through reduced interferon production, impaired antigen presentation, and decreased cytotoxic T lymphocyte infiltration [87]. Limited in vitro evidence suggests EDCs may enhance HPV E6 and E7 oncogene expression through epigenetic regulation wherein EDCs alter methylation patterns at HPV regulatory regions, potentially relieving transcriptional repression of viral oncogenes, alongside cellular signaling wherein BPA activates pathways including PI3K/AKT and MAPK that promote E6/E7 expression [65]. HPV E6 inactivates p53, representing the genomic guardian impairing DNA damage responses, with EDC-induced oxidative stress combined with p53 inactivation creating conditions for mutation accumulation [88].

## 9. Common Mechanistic Themes and Clinical Implications

Table 4 summarizes convergent molecular mechanisms across gynecological malignancies alongside corresponding therapeutic and prevention implications.

Despite tissue-specific differences, several molecular mechanisms recur across EDC-associated gynecological cancers, suggesting convergent pathways worthy of therapeutic targeting. EDCs rarely show absolute receptor selectivity; BPA binds ERα, ERβ, GPER1, androgen, progesterone, and thyroid hormone receptors with varying affinities [73]. This promiscuity enables simultaneous engagement of multiple receptors, amplifying proliferative signals through MAPK, AKT, and STAT3. Extensive receptor crosstalk—ER-AR, ER-thyroid hormone, and ER-growth factor receptor interactions—produces tissue-specific outcomes determined by coregulator expression, receptor isoform ratios, and chromatin accessibility [90].

DNA methylation, histone modifications, and microRNA alterations recur across breast, endometrial, and ovarian cancers, with developmental exposure establishing aberrant patterns that persist throughout life and create latent cancer predisposition [91]. Animal studies demonstrate parental EDC exposure affects offspring cancer risk through germline epimutations, with limited human evidence suggesting similar possibilities [93]. Epigenetic marks are reversible using DNMT inhibitors (azacitidine) or HDAC inhibitors (vorinostat), offering potential prevention strategies.

Oxidative stress represents the most universal EDC mechanism across breast, endometrial, ovarian, and cervical cancers, with elevated 8-oxoGua and lipid peroxidation markers consistently observed in EDC-exposed individuals across all four sites [21,26]. The cross-tissue conservation of ROS-mediated genomic instability—detailed mechanistically in Section 4.3—supports antioxidant biomarker panels as candidate cross-cancer screening tools, though clinical validation remains limited [22].

The Warburg effect (aerobic glycolysis) characterizes cancer cells across tissue types [27,28]. EDCs promote this metabolic shift: BPA, phthalates, and PFAS upregulate glucose transporters (GLUT1, GLUT3) and glycolytic enzymes (HK2, PFK, LDHA), with lactate accumulation acidifying the tumor microenvironment to promote invasion and immune evasion [94]. Chronic inflammation is both cause and consequence of cancer development, mediated through pro-inflammatory cytokines (IL-1β, IL-6, IL-8, TNF-α, COX-2), PD-L1 upregulation promoting T-cell exhaustion, and myeloid-derived suppressor cell recruitment creating immunosuppressive microenvironments [96].

Cancer stem cell populations exhibit enhanced treatment resistance and metastatic potential. EDC-induced stem cell enrichment provides mechanistic links to aggressive disease through stemness marker upregulation (Oct4, Sox2, Nanog, CD44, CD133) and dysregulated asymmetric division. Stem-cell-targeting strategies using Notch and Wnt inhibitors may help prevent EDC-associated cancers [23].

### 9.1. Clinical Translation and Counseling Recommendations

The mechanistic convergence of EDC effects across gynecological malignancies, combined with epidemiological evidence, necessitates practical clinical approaches for patient counseling and risk reduction. Table 5 provides evidence-based recommendations for different patient populations.

### 9.2. Diagnostic Implications and Biomarker Development

Integration of EDC exposure assessment into existing gynecological cancer diagnostic and screening workflows represents an emerging frontier in precision medicine. Current screening modalities—mammography for breast cancer, transvaginal ultrasound and CA-125 for ovarian cancer, endometrial biopsy for endometrial cancer, and Pap cytology with HPV co-testing for cervical cancer—are not designed to incorporate environmental exposure history. Mounting evidence supports adding EDC exposure data to multifactorial risk-prediction models that already integrate genetic, reproductive, hormonal, and lifestyle variables. The Tyrer-Cuzick and BOADICEA models for breast cancer risk could be augmented with biomarker-based EDC exposure indices, particularly for women without identified BRCA mutations who develop early-onset, ER-positive disease attributable in part to environmental exposures [5,61].

Diagnostic biomarker development for EDC-related cancer risk operates at three complementary levels. First, exposure biomarkers: direct measurement of EDCs and their metabolites in serum, urine, adipose tissue, breast milk, and meconium provides quantitative assessment, using gas chromatography-mass spectrometry, liquid chromatography-tandem mass spectrometry, and immunoassays. Persistent compounds (PCBs, organochlorine pesticides, PFAS) require serum or adipose measurement, whereas non-persistent compounds (BPA, phthalates) require urinary metabolite quantification with appropriate sampling frequency given short biological half-lives. Second, effect biomarkers reflecting downstream consequences include oxidative stress markers (8-OHdG, F2-isoprostanes), epigenetic alterations (DNA methylation, microRNAs), and inflammatory mediators (hsCRP, IL-6, TNF-α). Third, susceptibility biomarkers—polymorphisms in CYP1A1, CYP1B1, GSTM1, GSTT1, and COMT—modify individual susceptibility, supporting pharmacogenomic-style risk stratification.

Emerging diagnostic technologies offer substantial potential for translating mechanistic insights into clinically actionable tools. Liquid biopsy approaches detecting circulating tumor DNA, cell-free methylated DNA, and tumor-derived extracellular vesicles may enable early detection of EDC-driven malignancies before clinical manifestation. Multi-omics integration of genomic, transcriptomic, proteomic, metabolomic, and epigenomic data—analyzed through machine learning—can identify molecular signatures distinguishing EDC-associated tumors from sporadic cancers. For ovarian cancer, where current biomarkers (CA-125 and HE4 within the ROMA algorithm) lack adequate sensitivity for early-stage disease, EDC-related epigenetic and proteomic panels may improve diagnostic performance. Methylation-based screening tests for endometrial and cervical cancer could similarly incorporate EDC-responsive loci.

Practical incorporation of EDC-informed diagnostics into clinical workflows requires several considerations. Sensitivity and specificity of exposure biomarkers vary with sample type, timing of collection, and analytical platform, necessitating standardized protocols. Single-time-point measurements may inadequately capture chronic, low-level exposures, supporting repeated measurements or composite indices. Cost-effectiveness must weigh diagnostic gains against laboratory expenses, particularly for population-wide screening. Communication of EDC exposure results requires careful framing to avoid undue alarm while motivating evidence-based exposure reduction—a challenge analogous to genetic risk disclosure. Despite these challenges, advances in analytical chemistry, multi-omics, and computational risk modeling position EDC-informed diagnostics for meaningful clinical translation within the next decade.

Beyond initial diagnosis and risk stratification, EDC exposure assessment carries prognostic and surveillance implications. Patients with documented high EDC exposures may warrant intensified surveillance, particularly during high-vulnerability stages such as perimenopause when hormonal transitions may unmask latent EDC-induced abnormalities. EDC exposure profiles may also predict treatment response: tumors arising in high-estrogenic-EDC contexts may show altered hormone receptor signaling that influences response to selective estrogen receptor modulators or aromatase inhibitors. Integration of pre-diagnostic biomonitoring with post-diagnostic molecular tumor profiling could enhance precision oncology approaches, though prospective validation remains essential before routine implementation.

### 9.3. Dose–Response Relationships, Critical Windows, and Future Directions

Traditional toxicology assumes monotonic dose–response curves wherein effects increase proportionally with dose. EDCs frequently violate this assumption, demonstrating U-shaped, inverted-U, or biphasic curves. Mechanisms include receptor downregulation at high doses, metabolic saturation of detoxification pathways, compensatory feedback responses activating only at higher doses, and concentration-dependent receptor subtype selectivity [11]. For example, BPA produces maximal proliferative responses in breast cancer cells at 0.1–10 nM but reduced effects at micromolar concentrations, preferentially activating membrane-initiated GPER1/mERα signaling at low doses but classical nuclear ERα at high doses, producing distinct transcriptional profiles. The 2024 Spanish endometrial cancer study similarly demonstrated stronger associations for moderate xenoestrogen exposures than for the highest exposures [61].

**Figure 3:**
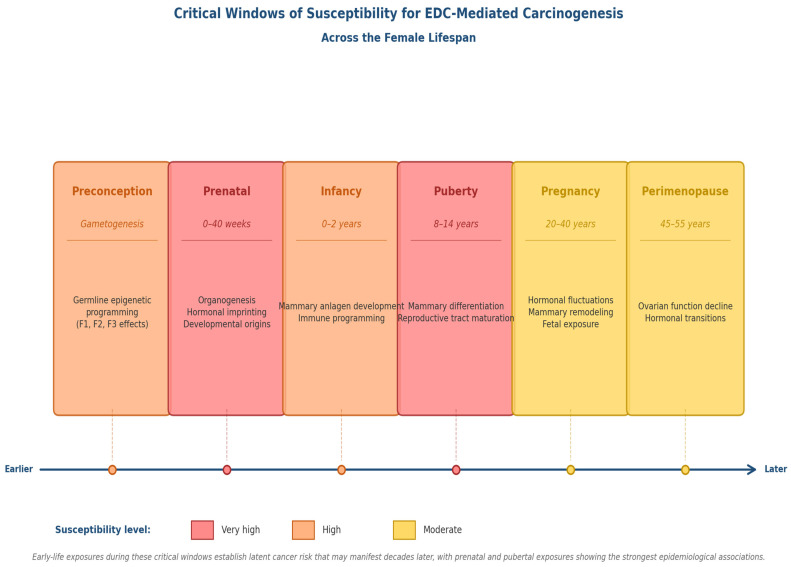
Critical windows of susceptibility for EDC-mediated carcinogenesis across the lifespan. The timeline illustrates periods of heightened vulnerability including preconception (germline epigenetic programming), prenatal development (organogenesis and hormonal imprinting, gestational weeks 0–40), infancy (early postnatal development, 0–2 years), puberty (mammary gland differentiation and reproductive tract maturation, 8–14 years), pregnancy (hormonal fluctuations and tissue remodeling, 20–40 years), and perimenopause (declining ovarian function, 45–55 years). Early-life exposures during these critical windows establish latent cancer risk that manifests decades later, with prenatal and pubertal exposures conferring particularly high risk for hormone-dependent malignancies. Latency periods of 20–50 years between exposure and cancer manifestation are indicated by timeline arrows connecting exposure windows to the typical age of cancer diagnosis.

As demonstrated in Figure 3, vulnerability to EDC-mediated carcinogenesis varies dramatically across the lifespan, with early-life exposures establishing latent cancer risk that manifests decades later.

Vulnerability to endocrine disruption varies dramatically across the lifespan, reflecting developmental processes uniquely sensitive to hormonal perturbation. During preconception and gametogenesis, parental EDC exposure establishes epigenetic marks in sperm and oocytes that are transmissible to offspring (DNA methylation at imprinted genes, histone modifications, small RNA populations); animal models demonstrate transgenerational effects through germline epigenetic inheritance, while DES granddaughter data suggest similar human possibilities [103]. Fetal organogenesis (weeks 3–8) is exceptionally vulnerable, affecting mammary gland primordia, Müllerian duct development, and ovarian follicle pool establishment, while later fetal growth (weeks 9–40) involves continued tissue differentiation [104].

Postnatal mammary development continues through childhood, with phthalate exposure linked to altered pubertal mammary development and BPA exposure to increased adolescent mammographic density. Puberty involves rapid tissue proliferation—ductal elongation, terminal end bud activity, endometrial gland expansion—during which DNA damage proves especially consequential and endogenous hormonal surges amplify EDC effects [105]. The CHDS cohort demonstrated strongest breast cancer associations for DDT exposure occurring before age 14 years. Pregnancy combines protective terminal differentiation (full-term pregnancy confers lifelong breast cancer protection) with vulnerable proliferation; first-trimester exposure may disrupt differentiation programs, whereas third-trimester exposure affects already-differentiated cells [97].

Cancer development requires decades, with latency periods between EDC exposure and clinical manifestation creating methodological challenges. The DES model involved prenatal exposure (1940s–1970s) with adenocarcinoma manifestation at ages 15–40 (1955–2010), representing a 15–40-year latency. The DDT model involved childhood exposure (1945–1972) with breast cancer manifestation at ages 40–70 (1985–2042), representing a 40–50-year latency [99]. Current BPA, phthalate, and PFAS exposures (2000s–2020s) may therefore manifest as cancer increases in the 2040s–2060s, complicating risk assessment.

Critical knowledge gaps persist across multiple fronts. Mixture toxicology requires urgent attention given that humans experience concurrent exposure to dozens of EDCs, yet research predominantly examines individual chemicals, failing to capture synergistic, antagonistic, or convergent pathway effects [13]. Priorities include realistic mixture studies designed using EDC combinations and concentrations reflecting actual human exposures, computational predictive models (QSAR and physiologically based pharmacokinetic models), and pathway-based grouping by shared mechanisms rather than structural similarity [100].

Multi-generational birth cohorts are essential for evaluating developmental EDC exposures and long-latency cancer outcomes. Requirements include biospecimen collection at critical windows (preconception, each trimester, birth, puberty, pregnancy), 40–50-year follow-up through cancer-relevant ages, three-generation designs enabling transgenerational assessment, and multi-omics integration of genomics, epigenomics, transcriptomics, proteomics, and metabolomics [102]. The costs and complexities are substantial but represent the only approach to definitively establish causality for developmental exposures.

Mechanistic biomarkers bridging exposure and disease are critical for early detection and prevention. Development requires identification through multi-omics studies of exposure-associated changes that predict cancer risk, validation in prospective cohorts demonstrating independent predictive value, clinical utility assessment determining whether biomarker-guided interventions reduce cancer incidence, and implementation considering cost-effectiveness, accessibility, and clinical workflow integration [106]. Candidate biomarkers include DNA methylation signatures, circulating microRNA profiles, oxidative damage markers (8-oxoGua), metabolomic fingerprints, and cancer stem cell populations.

Transgenerational effects—inheritance of EDC-induced phenotypes across generations—require mechanistic elucidation distinguishing direct exposure (F0), transplacental exposure (F1), and true transgenerational inheritance (F2 and beyond) through germline epigenetic mechanisms [103]. Animal studies demonstrate three-generation effects following ancestral EDC exposures, with the F3 generation showing increased cancer susceptibility, obesity, and reproductive abnormalities despite no direct exposure. Human evidence remains limited, but DES granddaughter studies suggest elevated F2 cancer risks warranting comprehensive investigation [98].

## 10. Conclusions and Integrated Synthesis

Across epidemiology, molecular mechanisms, and animal models, the accumulated evidence supports a contribution of endocrine-disrupting chemicals to gynecological malignancy through convergent pathways, with certainty varying markedly by cancer site. Breast cancer carries the strongest and most consistent evidence, with early-life DDT/DDE exposure showing the most robust associations (hazard ratios of 2–4-fold) and congener-specific PCB, phthalate, and PFAS findings requiring confirmation. Endometrial cancer shows compelling associations with xenoestrogen mixtures, although the reported non-monotonic dose-response requires cautious interpretation given residual confounding and limited power. Evidence for ovarian and cervical cancer remains emerging to limited (GRADE: Very Low), constrained for cervical cancer by difficulty separating heavy-metal effects from smoking. The recurring mechanistic themes—receptor promiscuity and crosstalk, epigenetic dysregulation with transgenerational potential, oxidative genomic instability, metabolic reprogramming, inflammation, and cancer stem cell enrichment—suggest that prevention and intervention strategies may be shared across sites. Together with non-monotonic dose-responses and the critical-window vulnerability detailed in Section 9.3, these findings represent a shift in toxicological thinking, while the multi-decade latency between developmental exposure and disease creates the central methodological challenge for establishing causality.

Individual exposure reduction is beneficial but insufficient given ubiquitous contamination, so population-level protection requires regulatory reform, including pre-market endocrine-disruption screening, chemical-class regulation, mixture risk assessment, and protection of vulnerable groups. Priority knowledge gaps include realistic mixture toxicology, multi-decade birth-cohort studies capturing critical-window effects, multi-omics biomarker development, and elucidation of transgenerational effects. For clinical practice, these data identify EDC exposure as a potentially modifiable cancer risk factor: counselling on exposure reduction during preconception, pregnancy, and early childhood is prudent, and integrating EDC exposure history into multifactorial risk-prediction and surveillance may refine individualized risk assessment, with multi-omics signatures offering a route to distinguish EDC-associated from sporadic tumors. Overall, the convergence of epidemiological, mechanistic, and clinical evidence supports a precautionary approach that minimizes exposures during critical developmental windows as a scientifically grounded public-health measure, while acknowledging that definitive causal confirmation awaits the prospective, multi-generational studies outlined above.

## Figures and Tables

**Table 1:** Classification of Major Endocrine-Disrupting Chemicals and Their Sources.

EDC Category	Representative Compounds	Primary Sources	Exposure Routes	Typical Biomarkers	Half-Life/Persistence
Bisphenol Compounds [29,30,31]	BPA, BPS, BPF, BPAF	Polycarbonate plastics, epoxy resins, thermal receipts, food can linings	Diet (93%), dermal, inhalation	Urinary BPA conjugates	6 h (rapid metabolism)
Phthalates [32,33,34]	DEHP, DBP, BBP, DEP, DiBP	PVC plastics, personal care products, medications, building materials	Diet (primary), dermal, inhalation	Urinary phthalate metabolites	Hours to days
Polychlorinated Biphenyls [35,36]	PCB-153, PCB-138, PCB-180	Legacy industrial uses, contaminated fish/dairy	Diet (>90% from animal fats)	Serum PCBs	Years to decades
Organochlorine Pesticides [37,38]	DDT/DDE, chlordane, dieldrin	Historical agricultural use, malaria vector control	Diet (contaminated foods), occupational	Serum pesticides	Years (highly persistent)
PFAS [39,40,41]	PFOA, PFOS, PFHxS, GenX	Food packaging, non-stick cookware, firefighting foam, water-resistant textiles	Contaminated water, diet, occupational	Serum PFAS	2–9 years (variable)
Heavy Metals [42,43,44]	Cadmium, arsenic, lead	Cigarette smoke, contaminated food/water, occupational	Inhalation, diet, water	Blood/urine levels	10–30 years (cadmium)
Parabens [45,46]	Methylparaben, propylparaben, butylparaben	Cosmetics, pharmaceuticals, food preservatives	Dermal, diet	Urinary parabens	Hours (rapid elimination)

**Table 2:** Molecular Mechanisms of EDC-Mediated Carcinogenesis in Gynecological Tissues.

Mechanism	Key Pathways/Processes	Representative EDCs	Primary Effects	Tissue-Specific Considerations
Estrogen Receptor Modulation [15,16,17]	ERα/ERβ activation, GPER1 signaling	BPA, DES, phthalates, alkylphenols	Enhanced proliferation, anti-apoptotic signaling	Breast and endometrium most sensitive; ERβ selectivity in ovary
Epigenetic Reprogramming [18,19,20]	DNA methylation, histone modifications, miRNA dysregulation	BPA, cadmium, phthalates, PCBs	Tumor suppressor silencing, oncogene activation	Developmental exposures establish persistent marks
Oxidative Stress [21,22]	ROS generation, DNA damage, lipid peroxidation	Heavy metals, PAHs, organochlorines	8-oxoGua formation, strand breaks, genomic instability	Compounded by impaired antioxidant defenses
Oncogenic Signaling [23,24,25]	PI3K/AKT, MAPK/ERK, STAT3, Wnt/β-catenin	BPA, phthalates, PFAS, DES	Proliferation, survival, metabolism, stemness	Pathway activation tissue-dependent
Metabolic Reprogramming [21,22,26,27,28]	Enhanced glycolysis, altered mitochondrial function	BPA, phthalates, cadmium	Warburg effect, lactate accumulation	Supports rapid proliferation
Inflammation [47,48]	IL-1β, IL-6, IL-8, TNF-α, COX-2, PGE2	PCBs, PAHs, heavy metals	Chronic inflammatory microenvironment	Promotes immune evasion
Stem Cell Enrichment [23,49]	Notch, Wnt, Hedgehog pathway activation	BPA, arsenic, phthalates	Expanded cancer stem cell populations	Enhances treatment resistance

**Table 3:** Key Epidemiological Evidence for EDC-Breast Cancer Associations.

EDC Category	Study Design	Sample Size	Key Findings	Effect Size	Critical Window
DDT/DDE [26,58,59]	CHDS prospective cohort	118 cases, 354 controls	Prenatal/childhood exposure	HR 3.7 (1.5–9.2) in utero (GRADE: Moderate)	Before age 14
PCBs [36]	Multiple cohorts	>12,000 pooled	Congener-specific; estrogenic PCBs	OR 1.3–1.6 highest exposure (GRADE: Low)	Peripartum period
BPA [66,67]	Multiple case-control studies	Variable	Inconsistent; adipose tissue levels stronger than urinary	OR 1.8 (1.1–3.0) adipose (GRADE: Very Low–Low)	Developmental
Phthalates [68,69]	Multiethnic Cohort	802 cases, 793 controls	DEHP metabolites; ER+/PR+ tumors	OR 1.45 (1.12–1.89) (GRADE: Low–Moderate)	Premenopausal
PFAS [70]	California Teachers Study	>60,000 women	Proximity to contaminated sites	Variable by compound (GRADE: Very Low)	Lifetime exposure

**Table 4:** Common Mechanistic Themes and Clinical Translation Opportunities.

Mechanism	Evidence Across Cancer Sites	Druggable Targets/Pathways	Prevention Strategies	Research Priorities
Receptor promiscuity [15,89,90]	Breast, endometrial, ovarian	Selective ER modulators, multi-receptor antagonists	Reduce EDC exposure during critical windows	Receptor crosstalk mapping
Epigenetic dysregulation [51,52,91]	All sites; transgenerational	DNMT inhibitors, HDAC inhibitors	Prenatal/early-life exposure reduction	Epigenetic biomarker panels
Oxidative stress [21,22,26]	All sites; synergizes with DNA damage	Antioxidants (controversial), ROS scavengers	Minimize heavy metal/PAH exposure	Oxidative damage biomarkers
Metabolic reprogramming [57,92,93]	Breast, ovarian; Warburg effect	Metformin, glycolysis inhibitors	Dietary interventions, exercise	Metabolomics profiling
Inflammation [47,94]	All sites; immune evasion	COX-2 inhibitors, anti-IL-6/IL-8	Anti-inflammatory diet, reduce smoking	Inflammatory biomarker panels
Stem cell enrichment [23,95]	Breast, ovarian; treatment resistance	Notch/Wnt/Hedgehog inhibitors	Developmental exposure prevention	Cancer stem cell markers

**Table 5:** Practical Recommendations for Reducing EDC Exposure and Gynecological Cancer Risk.

Population	Primary EDCs	Key Recommendations	Supporting Evidence
Pregnant women	BPA, phthalates, PCBs, PFAS [29,32,35,39]	Avoid plastic food containers, minimize canned foods, prefer glass or stainless steel over plastics generally (noting that “BPA-free” substitutes such as BPS and BPF may be equally or more potent endocrine disruptors [30,31]), consume low-mercury fish, use glass or stainless-steel containers, avoid thermal paper receipts	Developmental programming during first trimester; transgenerational effects demonstrated [95,97,98]
Women with genetic risk (BRCA1/2)	DDT/DDE, PCBs, dioxins [26,58,71]	Minimize persistent organochlorine exposure, choose organic produce for high-pesticide items, avoid thermal receipts, use glass/stainless steel storage, reduce animal fat consumption	Gene-environment interactions amplify risk; enhanced surveillance needed [4,89]
Adolescents (puberty)	Phthalates, parabens, BPA [32,45,68]	Limit personal care products, avoid fragranced items, choose paraben-free cosmetics, minimize plastic beverage bottles, reduce processed foods	Pubertal mammary development particularly vulnerable to endocrine disruption [10,69,70]
Women with endometriosis	Dioxins, PCBs, phthalates [20,35,37]	Reduce animal fat intake, choose organic dairy, minimize plastic food wrap, filter drinking water, avoid reheating food in plastic	Dioxin-endometriosis association established; increased cancer surveillance recommended [37,62]
Occupational exposures	Pesticides, solvents, metals [37,42,43]	Personal protective equipment use, workplace monitoring, job modifications during pregnancy planning, regular biomonitoring, post-shift hygiene	Occupational standards essential; document exposures for risk assessment [86,99]
Women with early menarche	BPA, phthalates, phenols [29,32,45]	Screen for metabolic syndrome, choose organic foods, avoid plastic storage, minimize personal care products, maintain healthy lifestyle	Early menarche linked to increased breast cancer risk; multiple interventions beneficial [69,91]
General population	Multiple EDC mixtures [13,100]	Mediterranean diet, minimize ultra-processed foods, use glass/stainless steel storage, choose fragrance-free products, filter water, regular cleaning, hand hygiene	Mixture effects exceed individual risks; holistic approach most effective [7,101,102]

The recommendations in Table 5 are evidence-informed, drawing on the epidemiological and mechanistic literature cited in the corresponding rows and throughout this review. Where direct interventional evidence that exposure reduction lowers gynecological cancer incidence is not yet available, the specific practical measures listed represent the authors’ expert opinion, derived from established exposure-source and biomonitoring data and applied within a precautionary framework. The supporting-evidence column indicates the principal sources underpinning each recommendation; it should not be read as implying randomized-trial confirmation of clinical benefit. Importantly, “BPA-free” labelling does not guarantee safety, as common substitutes (BPS, BPF, BPAF) exhibit comparable endocrine activity [30,31].

## Data Availability

No new data were created or analyzed in this study. Data sharing is not applicable to this article.
